# Leicester Royal Infirmary

**Published:** 1920-04-24

**Authors:** 


### LEICESTER ROYAL INFIRMARY.

### 147TH ANNIVERSARY MEETING.

Gratitude for the past and optimism and progress for the future marked the largely attended and influential Annual Meeting of the above institution; the evident interest was testimony to the fact that the institution is administered on democratic lines, with every section of the community interested, and sharers, by representation, in the control of the affairs and policy. The chair was taken by Sir Arthur Hazelrigg, Bart., who was congratulated by Mr. T. Fielding Johnson, jun., the Chairman of the Board, on the fact that he was taking that day a position his grandfather occupied, as Chairman of the institution, for a period of twenty-seven years.

In moving the adoption of the report, Mr. Fielding Johnson, jun., dealt with the satisfactory financial position, which he thought a cause for very real gratitude. But the future was more important than the past, and he pointed out that the institution was in need of more beds; if more beds were supplied, they must have more nurses. Additional beds were wanted, not only to admit more patients, but to admit them without delay, and, what was perhaps an even more important factor, that patients might remain in hospital a little longer than was possible in present circumstances.

They were not without hope that the important problems of the future might be tackled without delay, and they were encouraged by generous gifts, amounting to £40,000, which had already been promised for various purposes. This was a good start, and he hoped the Governors and subscribers would that morning agree to the Board proceeding at once with a new wing on the south-west side of the institution, capable of accommodating approximately 100 beds, and also with the enlargement of the Nurses' Home to accommodate some sixty additional nurses, the first important section of a new enlargement scheme.

The Board were also anticipating that the Leicester Frith Hospital for soldiers suffering from shell-shock would be vacated shortly by the Ministry of Pensions; and Sir Jonathan North, during whose mayoralty and under whose auspices the building was secured and placed at the Ministry's disposal, was hopeful of the possibility of the institution being administered by the infirmary, not as a convalescent home, but as a convalescent hospital for some eighty patients. Such a home would enable patients to be moved into the country when acute symptoms had subsided, or a few days after operative treatment when patients were on the way to convalescence.

Mr. C. J. Bond, C.M.G., Deputy Chairman, instanced the desire of the Government to maintain and keep all the good points of the voluntary system, and he indicated that the institution, which was well fitted, well equipped, and well organised was the one which could best negotiate if any question of Government help were raised. The High Sheriff of Leicestershire and Sir Arthur Hazelrigg, Bart., also spoke in support; the latter hoped that the Board would not lose sight of the desirability of providing paying wards. It was a source of satisfaction that those who could not afford medical attention when struck with sickness were provided with the best of medical and surgical attention and the best of nursing without money and without price, but there were many who, through poverty, were unequal to the strain involved in treatment in a nursing home who would willingly contribute according to their means. The report was unanimously adopted.

On the resignation of Mr. Alfred Corah, J.P., Treasurer, on the termination of three years of office, the Chairman of the Board alluded to the great work he had accomplished, and to his magnanimity as Treasurer in presenting to the institution a Charter of Incorporation. Mr. Corah's efforts as Treasurer had resulted in an increased income of several thousand pounds per annum. Sir Jonathan North, in proposing a vote of thanks, made eulogistic reference to the Treasurer's services, and complimented him on the basis on which his effort was organised.

Tribute to the careful attention and to the skill of the honorary medical and surgical staff was paid by Mr. A. W. Faire, C.B.E., Director of the Local V.A.D., and by Mr. G. A. Mitchell, who referred to the fact that one of his children, when almost a skeleton, received treatment in the institution, and exhibited with joy a present-day photographic enlargement as a tribute to the attention she received.

A resolution endorsing the enlargement scheme and suggesting that the Board should carefully consider present and future needs, with a view to a special meeting being called for the consideration of any required accommodation, and of ways and means of providing it, was moved by Sir Thomas Cope, Bart., Chairman of the County Council, supported by the High Sheriff of Rutland.

